# Repeated BOLD-fMRI Imaging of Deep Brain Stimulation Responses in Rats

**DOI:** 10.1371/journal.pone.0097305

**Published:** 2014-05-13

**Authors:** Tzu-Hao Harry Chao, Jyh-Horng Chen, Chen-Tung Yen

**Affiliations:** 1 Department of Life Science, National Taiwan University, Taipei, Taiwan; 2 Interdisciplinary MRI/MRS Lab, Department of Electrical Engineering, National Taiwan University, Taipei, Taiwan; University Medical Center Goettingen, Germany

## Abstract

Functional magnetic resonance imaging (fMRI) provides a picture of the global spatial activation pattern of the brain. Interest is growing regarding the application of fMRI to rodent models to investigate adult brain plasticity. To date, most rodent studies used an electrical forepaw stimulation model to acquire fMRI data, with α-chloralose as the anesthetic. However, α-chloralose is harmful to animals, and not suitable for longitudinal studies. Moreover, peripheral stimulation models enable only a limited number of brain regions to be studied. Processing between peripheral regions and the brain is multisynaptic, and renders interpretation difficult and uncertain. In the present study, we combined the medetomidine-based fMRI protocol (a noninvasive rodent fMRI protocol) with chronic implantation of an MRI-compatible stimulation electrode in the ventroposterior (VP) thalamus to repetitively sample thalamocortical responses in the rat brain. Using this model, we scanned the forebrain responses evoked by the VP stimulation repeatedly of individual rats over 1 week. Cortical BOLD responses were compared between the 2 profiles obtained at day1 and day8. We discovered reproducible frequency- and amplitude-dependent BOLD responses in the ipsilateral somatosensory cortex (S1). The S1 BOLD responses during the 2 sessions were conserved in maximal response amplitude, area size (size ratio from 0.88 to 0.91), and location (overlap ratio from 0.61 to 0.67). The present study provides a long-term chronic brain stimulation protocol for studying the plasticity of specific neural circuits in the rodent brain by BOLD-fMRI.

## Introduction

Functional MRI (fMRI) is an excellent tool for examining brain function, particularly the brain’s global spatial activation pattern [1]–[3]. Studies using rodent models have recently used fMRI to investigate adult brain plasticity after sensory deprivation [4], peripheral nerve injuries [5]–[7], and strokes [8], [9]. To date, most rodent fMRI studies have used the forepaw electrical stimulation model [10]–[13] or the whisker mechanical stimulation model [14]–[17] to acquire fMRI data, using α-chloralose as an anesthetic.

These current methods have limitations. Although α-chloralose is satisfactory in preserving hemodynamic activity and a strong BOLD response can be observed in animals anesthetized with α-chloralose [18]–[20], this anesthetic has several limitations regarding its use in longitudinal studies: (1) it requires animals to be catheterized for continuous intravenous (i.v.) administration, because intraperitoneal (i.p.) injections of α-chloralose cause severe inflammatory reactions in the rat [21]; and (2) repeated administrations of α-chloralose may cause hepatic and renal damage. Therefore, α-chloralose can be used only in terminal studies [22]. Brain plasticity studies using α-chloralose-based rodent fMRI can only compare the difference between sham and experimental groups, and are unsuitable for following brain changes over time in the same animal. Only one group had challenge this concept, claiming that with careful application of α-chloralose through tail vein, animals were recoverable for repeated BOLD-fMRI study [23].

Moreover, peripheral stimulation evokes limited BOLD responses in the major sensory pathways of anesthetized rats. Consequently, such a protocol is limited in the number of brain regions that can be studied. For example, traditional rodent fMRI protocols, using peripheral nerve deafferentation to study brain plasticity, are only able to examine the response of the intact side of the somatosensory afferent [5]–[7]. Previous rodent fMRI research has demonstrated that functional connections in the brain can be revealed using deep brain stimulation (DBS); such connections include the corticocortical connection between rat motor cortices (using unilateral motor cortex electric stimulation) [24], the thalamocortical pathways (using direct stimulation of medial thalamus [25] or VP thalamus [26]), and the perforant pathway to the rat hippocampus (using direct stimulation of the medial perforant pathway) [27]–[30]. These cases show that using DBS and fMRI together, researchers can effectively reveal spatial activation patterns in specific brain circuitry. However, the use of DBS-fMRI is still limited to acute study in these cases. One previous study use chronically implanted carbon fiber-based electrode for brain stimulation, but acute studies of fMRI were taken [31].

For longitudinal fMRI study in rodents, several groups have explored possible alternatives. Isoflurane is a common anesthetic for longitudinal study and can be used for fMRI study of forepaw stimulation in rat at very low concentrations [32]. It also has been shown useful for DBS fMRI studies [33], [34]. However, isoflurane is a strong vasodilator for cerebrovascular system, and possibly due to the high basal cerebral blood flow induced, dosage higher than 1 minimum alveolar concentration (MAC) of isoflurane tend to suppress the BOLD response [32], . In recent years, medetomidine is gaining in popularity in rodent fMRI study, because it is also suitable for longitudinal studies [37]. Rat anesthetized by isoflurane and medetomidine express similar BOLD signal change (2%–4%) to forepaw stimulation [37]–[39], but using medetomidine shows wider range of acceptable anesthetic depths. Studies using medetomidine-based rodent fMRI regarding functional recovery after strokes have already been conducted [40], [41]. Medetomidine is an α2-adrenoreceptor agonist, and its active enantiomer, dexmedetomidine, is also used in human medicine. Medetomidine and dexmedetomidine induce reliable sedation, analgesia, muscle relaxation, and anxiolysis [42]. These effects can be quickly reversed using atipamezole, an α2-antagonist.

A long-term, chronic brain stimulation model is useful for studying plasticity in adult rodent brains. In addition to longitudinally following the brain response to DBS, chronic implantation allows tissue healing around the electrode, and avoids the electrode from moving related to animal during fMRI study [31]. In this study, we used a dexmedetomidine-based fMRI protocol combined with chronic implantation of MRI-compatible stimulation electrodes in the thalamus to longitudinally follow thalamocortical responses in the rat brain. Using electric stimulation on the VP thalamus of dexmedetomidine-sedated rats, we demonstrated stable BOLD responses in the S1 cortex that were highly conserved in activation time courses and activated voxel numbers for at least 7 days. This protocol will enable rodent fMRI studies to be conducted regarding the long-term plasticity of specific brain circuitry in normal and diseased states.

## Materials and Methods

### Subjects

This study used 18 male Long-Evans rats (Rattus norvegicus; National Laboratory Animal Center, Taiwan), aged 8–10 weeks, and weighing 270–350 grams. Vendor health reports indicated that these rats were free of known viruses, bacteria and parasites. In the study, n refers to number of animals. Ten animals were used in DBS-fMRI study. Among the 10 animals, 4 animals were used in the preliminary study using isoflurane as the anesthetic. The other 6 animals were used in the dexmedetomidine anesthetized DBS study; one of these 6 animals dropped its stimulation electrode headpiece after the first day of fMRI experiment; another animal was additionally performed the isoflurane-based fMRI tests, and 5 of the 6 animals completed the repeated BOLD-fMRI experiments. Additionally, 8 animals without the electrode implantation were used to measure the control SNR of the unstimulated brain areas. All animals were kept in a temperature-controlled room (22°C), using a 12-h light/dark cycle. Food and water were available ad libitum. Prior to surgery, the animals were housed pairwise in type 3H cages filled with C-grade Sani-Chips (absorbent animal bedding). The Institutional Animal Care and Use Committee of National Taiwan University approved all experimental procedures. This study adhered to guidelines established by the Council of Agriculture (Taiwan) for the experimental use of animals.

### Animal Preparation

After the rats were anesthetized using sodium pentobarbital (50 mg/kg, i.p.), bipolar tungsten stimulus electrodes were implanted into their right VP thalamus (AP: −2.7 mm, ML: 2.5 mm, DV: 5–6 mm) with a receptive field in the left forepaw. During the implantation, thalamus receptive field was identified by the evoked spike signals when tapping the rat’s left forepaw. The neural signals were recorded from the implanted electrode, and amplified by a Grass amplifier (Model P5) with a high- and low-frequency cutoff of 3000 and 300 Hz, and a gain of 10000. The amplified signals were then play out of a Grass AM8 audio monitor for the identification of neuronal action potential signals. During surgery, rectal temperatures were measured and maintained at 37.5°C by a feedback-controlled heating pad. After the electrodes were implanted, animals receive ibuprofen (Yung Shin Pharm. Ind. Co. Ltd, Taiwan; 15 mg/kg/day, i.p. for 5 days) for pain relief, and lincomycin (Lita Pharmacy Co. Ltd, Taiwan; 30 mg/kg/day, i.m. for 5 days) for the prevention of infection. The animals were allowed more than 1 week recovery period before conducting the BOLD-fMRI experiment.

**Figure 1:**
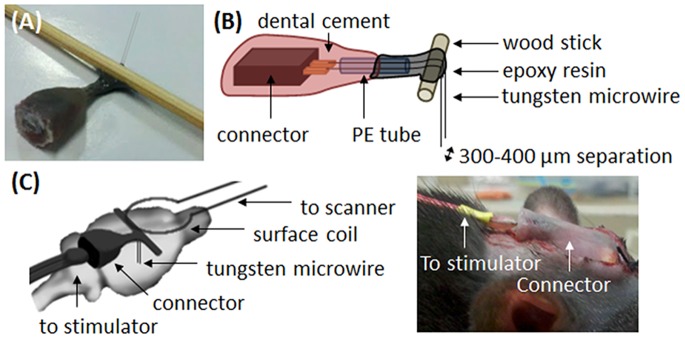
Details of the electrode set used in the current study. (A) Photograph of the electrode set used in the current study. (B) Schematic diagram with details of the structure inside the electrode set. Two tungsten wires were welded to the copper tip of the connector, and extended approximately 5 mm through the PE tube. The connector and PE tube were fixed together with dental cement, and the extended tungsten wires were bent 90^o^ around the wood stick and fixed using epoxy resin. The final length of the electrode wires, extended from the wood stick, was approximately 7 mm, and the distance between the 2 electrodes wires was approximately 300–400 µm. (C) The electrode was bent 90° to allow the placement of the MRI surface receiver coil over the rat head.

The stimulation electrode set was fabricated in the laboratory by aligning 2 Teflon insulated tungsten microwires (36 µm, product # M219930, California Fine Wire Co., Grover Beach, CA, USA) in parallel (separated by 300–400 µm), only the cut end of the electrode tips were exposed. The electrodes were bent 90° and laid flat along the rats’ skulls to allow the placement of the MRI surface receiver coil over their heads. The length of the electrode wires extended from the bending point to the brain target was about 7 mm. The connectors and the wire sets were fixed in place by dental cement, which were placed far from the recording sites to minimize signal distortion resulting from the metal in the connectors (Fig. 1). The impedance of the electrodes measured at 1 kHz and 100 Hz was approximately 145 kΩ and 860 kΩ respectively.

### BOLD-fMRI Acquisition

**Figure 2:**
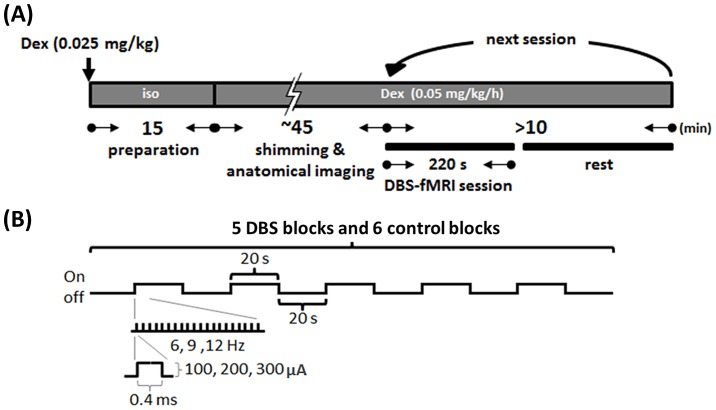
Experimental protocol. (A) Protocol for MRI scanning. For dexmedetomidine (Dex) based fMRI, the animal was first anesthetized using 5% isoflurane (Iso), then received a bolus of 0.025 mg/kg Dex subcutaneously. Iso was stopped 15 min later, and a continuous subcutaneous infusion of Dex (0.05 mg/kg/h) was initiated 15 min later for maintenance throughout the MRI experiment. The first session of the fMRI test was performed 60 min after the first bolus of Dex application. Each session of the fMRI test was separated by an inter-session interval of at least 5 min. (B) Stimulation protocol for DBS-fMRI session. Monophasic constant electrical current pulses with 0.4 ms duration, and the intensity and frequency adjusted in variable values, ranging from 100 to 300 µA and 6 to 12 Hz, were given by the block design, including 5 DBS blocks (stimuli on) and 6 control blocks (stimuli off), each block lasted 20 s.

All fMRI experiments were performed in the dark phase, between 1900 and 0700. Animals were anesthetized initially using 5% isoflurane, and maintained by 2% isoflurane while the animal was prepared for MRI scanning. In isoflurane-based fMRI experiments, isoflurane was then adjusted to 1–1.3% for the entire scanning period. In dexmedetomidine-based fMRI experiments, a bolus of dexmedetomidine (0.5 mL; 0.025 mg/kg; Dexdormitor, Orion, Espoo, Finland) was injected subcutaneously, and the isoflurane was discontinued after 15 min. The face masks were removed to allow the animals to spontaneously breathe room air. A continuous subcutaneous infusion of dexmedetomidine (1 mL/h; 0.05 mg/kg/h) was initiated 15 min after the bolus injection for the entire scanning period (Fig. 2A). The anesthesia procedures for isoflurane-based fMRI experiments and dexmedetomidine-based fMRI experiments were designed in reference to Kim, Masamoto et al. (2010) and Weber, Ramos-Cabrer et al. (2006), respectively.

During the MRI scan, rectal temperature was measured using a thermocouple (Model 1025, SA Instruments, Inc., New York, NY, USA), and maintained at 36.5–37.5°C using a circulated hot water bed. Respiration was monitored using a pressure sensor placed below the abdomen, and was stable at the range of 45–55 breath/min under isoflurane and 70–90 breath/min under dexmedetomidine anesthesia. The imaging data were acquired using a 7 Tesla scanner with a bore 30 cm in diameter (Bruker Biospec 7030 USR, Ettlingen, Germany). The system was equipped with a 670 mT/m (175-µs rise time) actively shielded gradient system (Bruker, BGA12-S) with an inner diameter of 116 mm. A receive-only surface coil was used to receive radio frequency signals, and a linear volume coil was used to transmit radio frequency pulses. The anatomical images were acquired using a RARE sequence (10 coronal slices, thickness = 1 mm, TR = 2500 ms, TE = 33 ms, matrix size = 160×160, FOV = 25×25 mm, average = 2). Before the BOLD-fMRI data acquisition, local shimming of the brain area was performed to improve the magnetic field homogeneity of the acquisition site (MAPSHIM; Bruker BioSpin). The BOLD-fMRI data were acquired using single shot gradient-echo echo planar imaging (EPI) (10 coronal slices, thickness = 1 mm, TR = 2000 ms, TE = 22 ms, matrix size = 80×80, FOV = 25×25 mm, bandwidth = 200 kHz).

An isolated stimulator (S48 Square Pulse Stimulator with Stimulus Isolation Unit, Grass Technologies, West Warwick, RI, USA) was used to deliver constant current pulses to the rats’ VP thalamus through the laboratory-fabricated MR-compatible bipolar electrode. These constant current pulses consisted of monophasic square wave electrical stimulation (0.4 ms, 100–300 µA, 6–12 Hz) divided into 5 blocks of 20-s-on/20-s-off cycles. The order for testing different stimulation parameters were randomized independently in each daily experiment of an animal. The randomized orders were obtained by using the MATLAB function- randperm(5), which gives a random permutation of the integers from 1 to 5. The numbers of 1 to 5 refer to 100 µA/9 Hz, 200 µA/9 Hz, 300 µA/9 Hz, 200 µA/6 Hz and 200 µA/12 Hz. Two additional scanning sessions were performed before the formal testing, and one additional scanning session was performed after the formal testing (with the stimulation parameter of 200 µA/9 Hz) to confirm the stability of the experimental condition during the whole daily experiment. In each scanning session, 10 dummy scans and 10 additional images for the baseline were acquired, resulting in a total of 120 images (Fig. 2B). The anode and cathode for bipolar stimulation of the 2 microwires separated by 300 microns were determined using whichever was most efficient to evoke greater S1 BOLD signal changes in each animal. All fMRI data were acquired between 1 and 2 h after the initial administration of dexmedetomidine.

### Histology

One day after collecting the final MRI scans, each rat with electrode implantation was euthanized by a sodium pentobarbital overdose (100 mg/kg, IP). Under deep anesthesia, a lesion for identification of electrode tip was produced by a constant 30 µA anodal current for 30 s through the electrode tip, and then perfused transcardially with saline followed by 4% formalin. The brain was removed and stored in a saturated sucrose solution overnight, then cut into serial coronal sections (50 µm). These sections were then processed for cresyl violet staining.

### BOLD-fMRI Data Processing and Analysis

Statistical activation maps were created using a statistical parametric mapping package (SPM8; www.fil.ion.ucl.ac.uk/spm). Prestatistics processing steps were applied to the raw data. These included: (1) Realigning and reslicing all EPI images within a time-series, based on their averaged image, to minimize movement artifacts; (2) Smoothing the images by using a Gaussian kernel with a full width at half maximum (FWHM) of 1 mm, which reduced white noise and blurred the images. Statistical analysis was conducted using general linear modeling with a hemodynamic response function. A 0.0078 Hz high pass filter was applied to remove the slow signal drift. To control the probability of false positive clusters below 0.05, we determined the significant BOLD response areas with an individual voxel threshold of P<.05 with the cluster size threshold of 114 continuous voxels according to the result from AlphaSim procedure [43].

The region of interest (ROI) analysis was conducted using the mean signal intensity of the supra-threshold voxels, located inside the anatomical defined S1 region, in T-map images. If there is no supra-threshold voxels inside the S1 region (which usually occurred when using a stimulation parameter below 200 µA, 9 Hz), then the ROI defined under the stimulation parameter of 200 µA, 9 Hz was used. This ROI then was used to obtain the time-series of the BOLD signal intensity from the realigned EPI images (excluding the dummy scans). The signal changes of the time-series were normalized to the first control block to determine the BOLD signal change as a percentage. The evoked response intensity was then calculated using the mean difference between the stimulated and resting conditions. The ROI analysis data are presented as the mean ± SEM. For the comparison of S1 activation under isoflurane and dexmedetomidine, since the S1 activation sizes in the isoflurane group of our study fluctuated session to session, to generate an average BOLD signal time-series, a conjunction analysis was applied. Briefly, an additional spatial registration of different fMRI sessions to a template T2 MR image was applied before the image-smoothing step, and the ROI analysis was only applied on the converged activation voxels in S1 region.

We measured the signal-to-noise ratios (SNR) of EPI in different brain areas, the ROI of different brain areas were manually drew within anatomical landmarks based on The Rat Brain Atlas of Paxinos & Watson [44]. These SNR values were collected from animals that had received tungsten electrode implants (Elec group, n = 10) and control animals that had not (Ctrl group, n = 8). We select S1 forepaw region as the cortical ROI, since we expect the BOLD response to VP stimulation should be observed there. We also select caudate putamen (CPu) and globus pallidus (GP) for represent subcortical structures. A 3×3 voxel square located below the electrode tip was select as another ROI to understand the signal distortion around the stimulation site.

### Reproducibility

To assess the reproducibility of S1 BOLD response acquired in week 1 and week 2, activation overlapping and size measures were employed [45], [46]. The overlap ratio of S1 activation area (R_ij overlap_) was defined as R_ij overlap_ = 2*V_ij overlap_/(V_i_+V_j_), in which V_ij overlap_ is the number of the S1 activation voxels which found in both week 1 and week 2. V_i_ and V_j_ are the total number of S1 activation voxels in week 1 and week 2 respectively. The ratio of S1 activation voxels observed in week 1 and week 2 (R_ij size_) was calculated by R_ij size_ = 2*V_ij smallest_/(V_i_+V_j_), where V_i_ and V_j_ are defined as above, V_ij smallest_ is the smallest of V_i_ and V_j_. Both indexes range from 0 (worst) to 1 (best).

### Statistical Analysis

Three primary outcome measures related to DBS-fMRI reproducibility were analyzed: (1) The consistency of each quantitative measurement (response amplitude and voxel numbers to different stimulation intensity) between week 1 and week 2. (2) The overlap ratio of S1 activation area and activation voxels observed in week 1 and week 2. (3) The differences of the BOLD response amplitudes or voxel numbers observed between week1 and week 2. In addition, two secondary outcome measures were evaluated: (1) SNR of EPI in different brain areas obtained from animals that had received tungsten electrode implants and control animals that had not. (2) Stimulation intensity- and frequency-dependent relationship of VP stimulation-S1 BOLD response measured using 0.4 ms-width pulses stimulation under dexmedetomidine anesthesia.

All data are expressed as mean ± standard error. For each test, the experimental unit was an individual animal. Test for normality was performed by Kolmogorov–Smirnov test. Significant differences of SNR between control (n = 8) and rats implanted with tungsten electrode (n = 10) were determined using Mann–Whitney U test with a 2-tailed distribution. Intraclass correlation coefficient (ICC) was used to understand the reproducibility of each quantitative measurement (response amplitude and voxel numbers to different stimulation intensity, for each stimulation intensity: n = 5) between week 1 and week 2. Significant differences of the BOLD response amplitudes or voxel numbers between stimulation parameters or between week1 and week 2 were determined using Kruskal-Wallis one-way analysis of variance (ANOVA) with Duncan’s new multiple range post-hoc test. *P* values <.05 were considered statistically significant.

## Results

**Figure 3:**
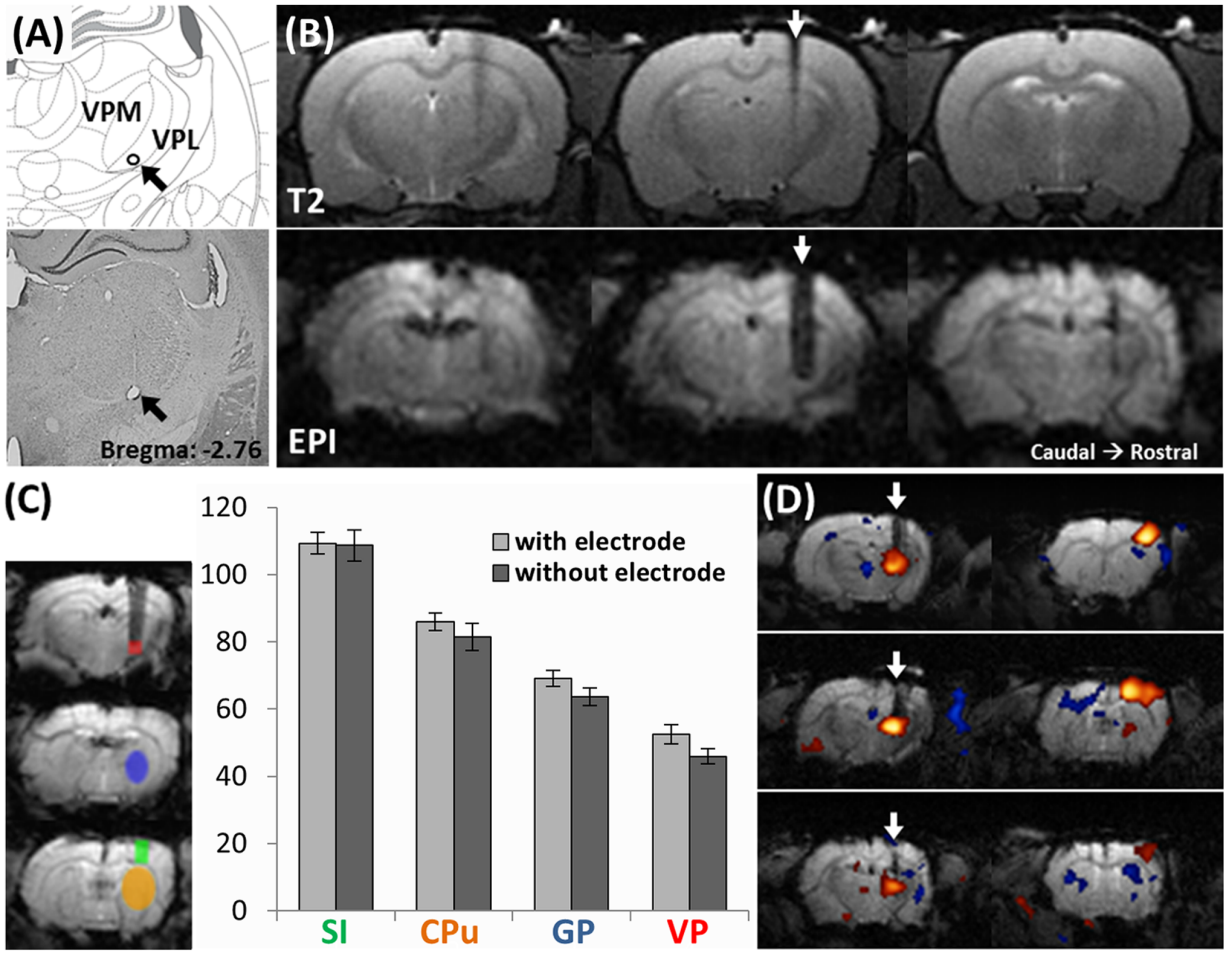
Evaluation of image distortion caused by tungsten electrode. (A) An example of the identification of the electrode target site by cresyl violet stained brain section. The lesion at the electrode tip (black arrow) was produced by a constant electric current of 30 µA for 30 s before euthanizing the animal. The electrode lesion was located within the forepaw region of the VPL thalamus. (B) The tungsten electrode caused limited distortion to the MR signal around the electrode in T2 anatomical images (upper panel) and in EPI images (lower panel). In each panel, 3 serial scans from caudal (left) to rostral (right) are shown. The white arrows indicate the electrode track. (C) SNR in different brain areas in control rats and rats implanted with a tungsten electrode. The ROIs were defined as shown in the left panel: VP thalamus = red, globus pallidus (GP) = blue, caudate putamen (CPu) = yellow and S1 = green. In all 4 brain areas, no significant difference between the tungsten electrode compared with the control was observed. (D) Representative DBS-fMRI results from 3 individuals. White arrows indicate the electrode tracks. Positive BOLD response can be clearly observed in the VP thalamus and ipsilateral S1.

The receptive field was electrophysiologically confirmed during electrode implantation. At the end of the study, to further confirm the electrodes were implanted in the forepaw region of the VP thalamus, the electrode tracks were visually confirmed in the cresyl violet stained brain sections (Fig. 3A).

We evaluated the distortion caused by the tungsten electrodes to the images. Figure 3B shows the representative T2 anatomical images (upper panel) and EPI images (lower panel) around the electrode. In each panel, 3 serial scans from caudal (left) to rostral (right) are shown; the middle image depicts the electrode implant site. The distortion caused by the tungsten electrode to the MR signal around the electrode site, in both the T2 anatomical images and the EPI images, was limited. SNRs were not observed to be significantly attenuated in S1 (Ctrl: 109±5, Elec: 109±3), CPu (Ctrl: 86±4, Elec: 81±3), or GP (Ctrl: 69±3, Elec: 64±2). SNRs below the tungsten electrodes were decreased (from an average of 53±2 to 46±3), but this was not statistically significant (*P* = .10, Fig. 3C). Figure 3D shows 3 representative DBS-fMRI results featuring one section taken from the thalamus level and another section taken from S1 level. The electrodes were implanted inside the VP thalamus; we observed that image distortion caused by the electrodes did not prevent the detection of functional BOLD responses within the VP thalamus, and a robust BOLD response was observed in S1 (Fig. 3D). These data suggest that the levels of distortion caused by the electrodes were acceptable and did not severely attenuate BOLD signal changes in the VP thalamus; the electrodes had no obvious influence on signals further in the rostral forebrain.

**Figure 4:**
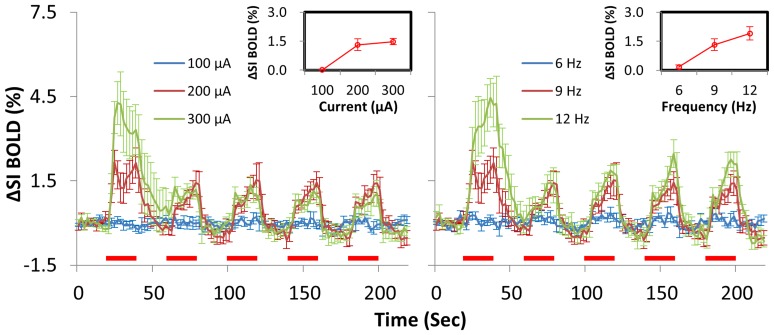
Amplitude and frequency dependency of BOLD response in dexmedetomidine-anesthetized rats. Averaged time courses within the S1 of 6 animals are plotted for 3 stimulus currents in the left panel, and 3 frequencies in the right panel. Red bars under the time courses indicate the 20-s stimulation period. The insets show the response amplitude versus the stimulus current or frequency.

To establish a baseline VP stimulation-S1 BOLD response relationship under dexmedetomidine anesthesia, we used 0.4 ms as the pulse width to test different stimulation amplitudes (100, 200, and 300 µA at 9 Hz) and frequencies (6, 9, and 12 Hz at 200 µA) during separate DBS-fMRI sessions and in random order (n = 6). Significant activated voxels in S1 revealed in each stimulation parameter were chosen for ROI analysis. We discovered both amplitude- and frequency-dependent thalamic-evoked BOLD responses in S1 (Fig. 4). Stimuli below 200 µA and frequency below 9 Hz were insufficient to evoke BOLD responses in S1. Stronger stimuli, over 300 µA or 12 Hz, only enhanced the BOLD response during the first stimulus block (Fig. 4), and often resulted in nonspecific brain activation or struggle motions in the animals.

**Figure 5:**
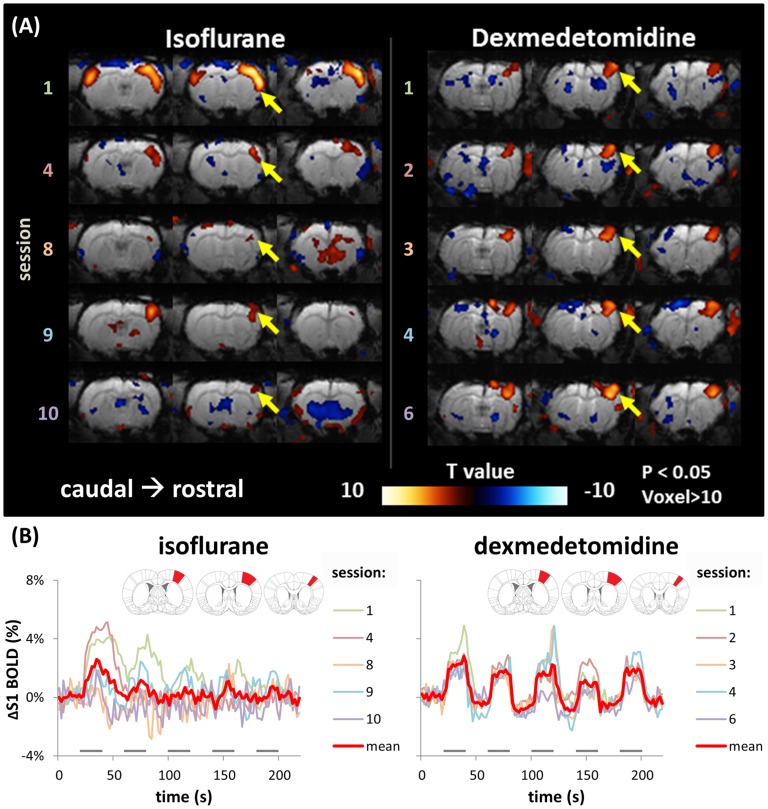
Stability of DBS-induced BOLD response under isoflurane and dexmedetomidine anesthesia among a series of sessions within a one-day experiment. Here shows the examples from one animal under isoflurane- and dexmedetomidine-based fMRI, tested on different days respectively. (A) The BOLD activation maps (*P<.05*, voxels >10) from a consequent of 5 repeated fMRI sessions (inter-scan interval: 10 min) with the same DBS parameter (0.4 ms, 200 µA, 9 Hz). When under dexmedetomidine anesthesia, a stable activation pattern was evident in S1 among sessions. Session 5 was eliminated because of an unexpected animal motion. When under isoflurane anesthesia, although a conserved positive BOLD response was observed in S1, the response pattern varied among sessions. In sessions 2, 3, and 5–7, different stimulation parameters were used, hence they are not shown in this figure. (B) The time courses within S1 for the 5 repeated scans were plotted together. The red trace is the average time course of all 5 scans, and the gray bars under the time courses indicate the 20-s stimulation period. Inset shows the S1-forelimb ROI used to obtain the signal time course. Variability was high under isoflurane anesthesia, compared with that under dexmedetomidine anesthesia.

The stability of DBS-evoked BOLD responses under dexmedetomidine anesthesia was ascertained by the same response pattern to the same stimulus parameters (200 µA, 9 Hz) in the first and the last session in all studies. Furthermore, in one rat, we performed 6 repeat sessions using the same stimulus parameters (200 µA, 9 Hz). In this rat, the spatial activation patterns of the BOLD responses were stable (Fig. 5A, right panel. The 5th session was eliminated, because of unexpected head motion during the scanning). The time courses of the BOLD signal changes between the sessions in the S1 activated area were also similar (Fig. 5B, right panel). The activated voxel numbers from the 1st to the 6th session (excepting the 5th session) were 141, 209, 166, 190, and 194, respectively (standard deviation (SD) = 23.9), and the BOLD response amplitudes were 2.58%, 2.99%, 2.43%, 2.96%, and 2.52%, respectively (SD = 0.23%). It also suggests 200 µA at 9 Hz was a good parameter for inducing robust (over 2%) and stable S1 BOLD response to VP stimulation under dexmedetomidine anesthesia in our condition.

In addition, because isoflurane is commonly used in animal MRI studies, and has been applied in certain rodent fMRI studies [32], [47], [48], we also evaluated whether isoflurane-anesthetized rats could yield stable BOLD responses when using our protocol. The left panel of Figure 5 shows the representative result from an isoflurane-anesthetized rat; these results were compared with dexmedetomidine results acquired on another day, using the same stimulus parameters (200 µA, 9 Hz). We allowed the rat to spontaneously breathe the anesthetic mixture, and the depth of anesthesia was controlled by maintaining the rat’s breath frequency at 55 to 60 breath/min. Rectal temperature was measured and maintained at 36.5–37.5°C during the fMRI procedure. We observed that the BOLD response pattern was extremely unstable. The response amplitude and voxel number in S1 fluctuated widely between sessions (Fig. 5A, left panel). The ROI analysis also showed no consistent patterns when using identical stimulation parameters (Fig. 5B, left panel).

**Figure 6:**
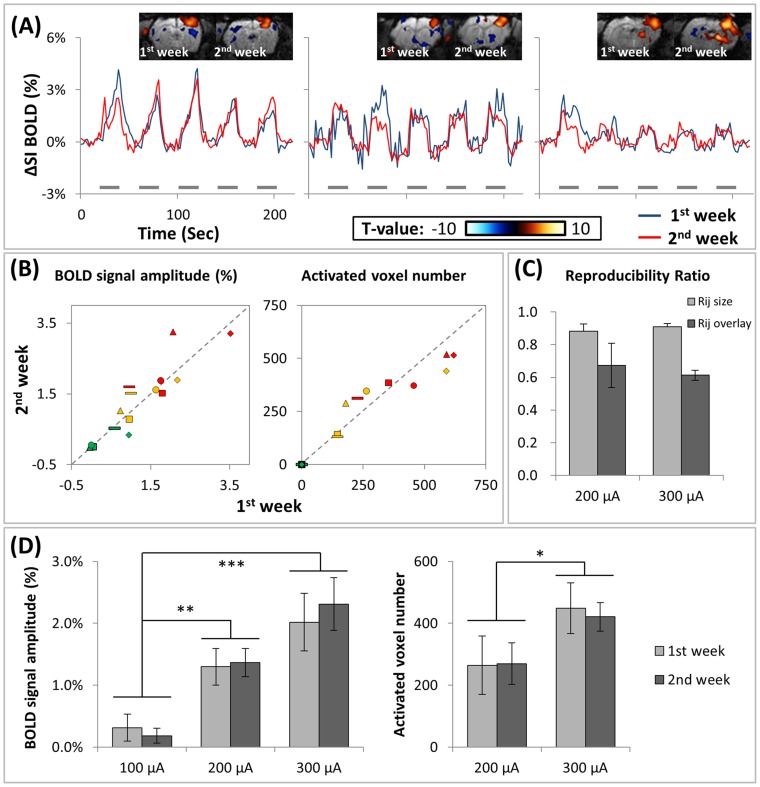
Reproducibility in repeated (separated by 5 days) fMRI tests of DBS-induced BOLD response under dexmedetomidine anesthesia. (A) Original data of longitudinal BOLD responses from 3 representative experiments. Each rat was scanned in 2 repeated sets of sessions with a 1-week interval. Gray bars under the time courses indicate the 20-s stimulation period. The stimulation parameter used was 200 µA with 9 Hz. Similar dynamic BOLD responses in S1 were observed between the session in Week 1 (blue trace) and the session in Week 2 (red trace). The activation map shows the spatial distribution of the BOLD response, the left and right maps show the results from the Weeks 1 and 2, respectively. (B) The response amplitude (left panel) and activated voxel number (right panel) in Weeks 1 and 2 were plotted against each other. All the stimulation parameters from each animal (n = 5) were pooled. Five individual rats were labeled as following symbols: •▴♦**–**▪. Different stimulation parameters were labeled as different colors (red: 9 Hz/300 µA, yellow: 9 Hz/200 µA, green: 9 Hz/100 µA). The cortex response amplitudes and activated voxel number in the 2 weeks showed high consistency. In the pooled data of different stimulation parameters: ICC = 0.9169 (*p<.0001*) for activation amplitude; ICC = 0.9569 (*p<.0001*) for activation voxel number. In each stimulation parameters: ICC = 0.82 (*p<.05*) and 0.73(*p<.05*) when using the stimulation parameters of 200 µA and 300 µA (9 Hz) for activation amplitude, and ICC = 0.89 (*p<.05*) and 0.82 (*p<.05*) when using the stimulation parameters of 200 µA and 300 µA (9 Hz) for activation voxel number. (C) Reproducibility of statistical parametric maps in different stimulation parameters. Overlay ratios ranged from 0.50 to 0.88 (mean = 0.67, SD = 0.14) and 0.59 to 0.66 (mean = 0.61, SD = 0.03) when using the stimulation parameters of 9 Hz/200 µA, 9 Hz/300 µA respectively. Size ratios ranged from 0.76 to 0.99 (mean = 0.88, SD = 0.04) and 0.84 to 0.96 (mean = 0.91, SD = 0.02) when using the stimulation parameters of 9 Hz/200 µA, 9 Hz/300 µA respectively. (D) The S1 BOLD signal amplitudes and activated voxel numbers under different stimulation parameters. The BOLD response amplitudes and activated voxel numbers were significantly different between stimulation parameters, but not between week 1 and week 2. (*p<.05, **p<.01, ***p<.001).

To test the long-term reproducibility of these results, and the applicability of this study to longitudinal research, each rat was scanned twice with an intersession interval of 7 days. Similar dynamic BOLD responses were observed between Sessions 1 (blue trace) and 2 (red trace) in S1, using the stimulation parameters of 200 µA and 9 Hz (Fig. 6). The S1 response amplitudes between Sessions 1 and 2 have strong consistency with an intraclass correlation coefficient of 0.82 (Fig. 6B, left panel, yellow data points, *P*<.05, n = 5) and 0.73 (Fig. 6B, left panel, red data points, *P*<.05, n = 5) when using the stimulation parameters of 200 µA and 300 µA (9 Hz) respectively. The activated area sizes in both sessions also have strong consistency, with an intraclass correlation coefficient of 0.89 (Fig. 6B, right panel, yellow data points, *P*<.05, n = 5) and 0.82 (Fig. 6B, right panel, red data points, *P*<.05, n = 5) when using the stimulation parameters of 200 µA and 300 µA (9 Hz) respectively. Overlap and size ratios between different stimulation parameters were similar. Size ratios ranged from 0.76 to 0.99 (mean = 0.88, SD = 0.04) and 0.84 to 0.96 (mean = 0.91, SD = 0.02) when using the stimulation parameters of 9 Hz/200 µA, 9 Hz/300 µA respectively. The overlay ratios were generally lower, which ranged from 0.50 to 0.88 (mean = 0.67, SD = 0.14) and 0.59 to 0.66 (mean = 0.61, SD = 0.03) when using the stimulation parameters of 9 Hz/200 µA, 9 Hz/300 µA respectively. Stimulation below 200 µA was usually not able to evoke any detectable S1 BOLD response, thus is not shown here (Fig. 6C). We used ANOVA to test whether the S1 BOLD response amplitudes and activated voxel numbers were different between stimulation parameters or between week 1 and week 2. The amplitude of activation in S1 in week 1 (week 2) comprised 0.31±0.22% (0.18±0.12%) when using the stimulation parameters of 9 Hz/100 µA, 1.30±0.30% (1.37±0.23%) when using the stimulation parameters of 9 Hz/200 µA, and 2.02±0.47% (2.31±0.42%) when using the stimulation parameters of 9 Hz/300 µA. The voxel number of activation in S1 in week 1 (week 2) comprised 264.4±93.9 (269.4±66.6) when using the stimulation parameters of 9 Hz/200 µA, and 449.0±81.9 (420.6±46.1) when using the stimulation parameters of 9 Hz/300 µA. We found significantly different S1 BOLD response amplitudes and activated voxel numbers between stimulation parameters, but not between week 1 and week 2 (Fig. 6D). These data suggest that chronic implantation of stimulation electrodes could enable robust, stable thalamus and S1-coupled BOLD responses over 7 days, using dexmedetomidine as an anesthetic.

## Discussion

The present study showed that distortion caused by conventional tungsten wire to EPI data was limited. Using dexmedetomidine as an anesthetic, it was possible to repeatedly image VP stimulation-evoked BOLD responses in the S1 over the course of a week in the same rat. BOLD responses in S1 were dependent on stimulation amplitude and frequency. Responses during the 2 sessions were comparable in amplitude and spatial distribution under all stimulation parameters tested; therefore, it was possible to longitudinally compare the stimulation-response relationship of specific brain circuits in the same individual. This study provides a reliable method for longitudinally following VP stimulation-evoked BOLD responses in the rat brain.

### Potential of DBS-fMRI for Studying Plasticity in Adult Rodent Brain

In our presented data, stable BOLD response amplitudes to brain stimulation were repetitively obtained (fig. 6), making longitudinally following the plasticity in certain brain circuitry by BOLD fMRI in rodent model possible. However, the response location seems intrinsically unstable (overlap ratio from 0.61 to 0.67), even in successive scan sessions, which can also be found in human studies [45], [46]. It may reflect the natural operation of the brain activity, and seems that a subtle plasticity in response location shift may be difficult to identify by BOLD fMRI. A recent study of LTP induced by high-frequency stimulation of the perforant path shows that about 20% increase in EPSP amplitude in dentate gyrus can produce significant increase of the BOLD response amplitude to perforant path stimulation, not only in hippocampal formation, but also nucleus accumbens, the anterior olfactory nucleus, and the perirhinal and prefrontal cortex. In hippocampal formation, the BOLD response enhancement paralleled EPSP enhancement, and in other areas, the BOLD responses were enhanced very little after LTP induced [30]. This shows that the response amplitude change in the DBS-fMRI can be used to detect brain plasticity.

### Tungsten Electrodes used in DBS-fMRI

Tungsten electrodes were used in this study because they are frequently used in electrophysiological studies, they are easy to obtain, and several studies have evaluated their use in combination with DBS-fMRI [24], [25], [27]. One DBS-fMRI study showed that tungsten electrodes caused severe EPI distortion, resulting in images that were unacceptable for analysis; however, a later study using tungsten electrodes to stimulate perforant pathways successfully evoked a robust BOLD response in rat hippocampi [27]. This difference may be explained by the different diameters of the tungsten electrodes used: the electrode used in the first study was 250 µm in diameter, whereas the tungsten electrode used in the second study was 114 µm in diameter. Using a thinner rather than thicker tungsten electrode may be crucial to ensure that the electrode does not distort the images. The tungsten electrode we used in this study was substantially thinner (36 µm) than those used in previous studies.

### Anesthesia in Longitudinal Rodent fMRI Study

Rodent fMRI using the anesthetic α-chloralose has been considered unsuitable for use in longitudinal studies because of its negative physiological side effects [21], [22], and only one report has claimed that a new, commercially available formulation of α-chloralose, administered carefully through the tail cannula, allowed for repeated fMRI studies of the same animal [23].

Medetomidine, which is primarily used for short-term procedures in veterinary medicine, has recently been considered for use in longitudinal rodent fMRI studies. Using forepaw electric stimulation and medetomidine as an anesthetic, studies have demonstrated a robust BOLD response in S1 reproducible over 5 days. In these studies, a bolus of 0.05 mg/kg, followed by a 0.1 mg/kg/h subcutaneous infusion was used [37], [38], [49]. Because dexmedetomidine is the dextrorotatory isomer of medetomidine, we assumed that dexmedetomidine was twice as effective as medetomidine. The dosage of dexmedetomidine used in this study was half of the dosage used in regular medetomidine protocols. In our study, all fMRI data were acquired between 1 and 2 h after the initial administration of dexmedetomidine. Our experience showed that 30 to 60 min were required for anatomical imaging and adjusting the MR machine for functional imaging. On the other hand, a constant infusion of medetomidine is not sufficient to maintain sedation for fMRI protocols longer than 3 h [38]. Moreover, a study reported that epileptic responses to forepaw stimulation appeared in runs at >2 h after medetomidine sedation [50]. We observed irregular prolonged S1 BOLD responses to VP thalamus stimulation in runs at >3 h after sedation, which is consistent with a previous study of cerebral blood flow (CBF) in S1 cortex responses to forepaw stimulation [50]. Therefore, 1–2 h is a reasonable time window for BOLD-fMRI studies using medetomidine protocols.

Isoflurane is another anesthetic that has often been considered for use in longitudinal fMRI studies. Previous studies have reported that S1 BOLD responses to electrical forepaw stimulation (1.5–2.0 mA) can be detected using isoflurane anesthesia at approximately 1 MAC [32], [47], [48] (isoflurane = 1.3±0.1%) in rats [51], [52]. We examined the BOLD response to VP stimulation in isoflurane-anesthetized (1.0%–1.3%) and spontaneously breathing rats. Although breathing rate, body temperature, heart rate, and blood pressure were all maintained within normal ranges during our fMRI study, consistent with previous studies [48], [53], BOLD responses were unstable between experimental sessions. Yielding stable BOLD responses in isoflurane-anesthetized rats may require mechanical ventilation to precisely control the rats’ physiological condition, because of the sensitivity of the neurovascular coupling property to isoflurane [54]. Moreover, we did not detect BOLD responses at >1.5% isoflurane. This is consistent with previous BOLD and CBF fMRI studies using forepaw stimulation in rats [48] and visual stimulation in cats [35], [36], which reported the optimized concentration for isoflurane anesthesia as 1.0%–1.25%.

### Electrical VP Stimulation Under Dexmedetomidine Anesthesia

Many studies regarding peripherally evoked BOLD responses in S1 have reported that frequency-dependent BOLD responses often plateau at a certain frequency. Using medetomidine, the maximum BOLD response elicited in S1 was approximately 9 Hz [49]; this is similar to the optimal frequencies of isoflurane (12 Hz) [32], [47] and enflurane (10 Hz) [55], whereas 1–5 Hz is the optimal frequency when using α-chloralose [13], [56]–[62]. In rodent fMRI studies of VP electrical stimulation evoked BOLD response in S1, 3 Hz had been reported as the optimal frequency when using α-chloralose as the anesthetic, which is similar to the frequency used for peripheral stimulation [26]. On the other hand, 25 Hz had been reported as the most efficient stimulation frequency for VP when using isoflurane as the anesthetic [33], which was about twice of the frequency used for peripheral stimulation. It seems that the relationship of the optimal stimulation frequency for peripheral and VP stimulation may vary under different anesthesia.

In our study, we used dexmedetomidine as the anesthetic, the BOLD responses in S1 evoked by direct thalamic stimulation were not saturated when the stimulation frequency exceeded 9 Hz, but continued to increase with frequencies up to at least 12 Hz (Fig. 4) at 200 µA. Under our experimental condition, using a stimulus frequency of 15 Hz seemed too strong, and often caused an irregular increase in respiration rate and an increase in motion. Similarly, the BOLD response increased with stimulation strength, but using a stimulation current above 300 µA caused animal motions. Note that stimulus frequency over 100 Hz is widely used in clinical DBS therapy, and also has been used in DBS fMRI study [34]. However, the 0.4 ms pulse width used in this study is relatively longer than the pulse width commonly used in clinical DBS application (around 0.1 ms), which could affect frequency-tuning measurement. Whether a shorter pulse width gives different frequency-tuning curve in dexmedetomidine-based fMRI needs further studies.

Additionally, increasing of the stimulation current to 300 µA or frequency to 12 Hz in our condition seemed only enhanced the response in the first stimulation epoch. It may reflect an adaptation effect involved in the operation of VP-S1 pathway. Previous study revealed the higher stimulation frequency lead to more rapid decreasing of S1 BOLD response to forepaw stimulation due to an adaptation effect under isoflurane-anesthetized condition [47]. The study of adaptation in olfactory system showed the adaptation of BOLD response in olfactory bulb is stimulation strength dependent, and caused long lasting suppressed BOLD response in later stimulation epochs [63]. Therefore, in our study, an adaptation effect may be a reason why the enhancing BOLD response was only observed in the first stimulation epoch, when increasing the stimulation current to 300 µA or frequency to 12 Hz. A longer resting period between stimulation epochs may help the recovery of VP-S1 system to baseline state.

### Significance

In this study, we used chronic teflon-coated tungsten bipolar-stimulation electrodes to investigate VP-S1 circuitry in the rat brain. We demonstrated a stable stimulus-amplitude- and frequency-dependent BOLD response in S1 in dexmedetomidine-sedated rats. These BOLD responses were reproducible over a week. This study provides a reliable technique for repeated tracing brain circuitry connectivities in rats. Brain circuitry plasticity is likely involved in many diseases, and our method is potentially useful for longitudinally following the development of these diseases inside the brain, to understand the neurobiological mechanisms of these diseases. In addition, DBS has been used to clinically treat a variety of neurologic conditions, including essential tremors, Parkinson’s disease, dystonia, Tourette’s syndrome, chronic pain, depression, and obsessive compulsive disorder [64]. However, the precise mechanism by which DBS acts remains unclear, and our method is also potentially applicable to the study of the neurobiological mechanism by which DBS acts, to optimize treatment parameters for these diseases.
